# Functional mobility and pain are improved for 6 years after adolescent bariatric surgery

**DOI:** 10.1002/oby.24285

**Published:** 2025-04-21

**Authors:** Neil Thivalapill, Todd M. Jenkins, Thomas H. Inge, Changchun Xie, Anita P. Courcoulas, Carroll M. Harmon, Michael A. Helmrath, Stephanie Sisley, Marc P. Michalsky, Justin R. Ryder, Teen‐Longitudinal Assessment of Bariatric Surgery (Teen‐LABS) consortium

**Affiliations:** ^1^ Department of Surgery Weill Cornell Medicine New York New York USA; ^2^ University of Cincinnati College of Medicine Cincinnati Ohio USA; ^3^ Department of Pediatrics Cincinnati Children's Hospital Medical Center Cincinnati Ohio USA; ^4^ Department of Surgery Northwestern University Feinberg School of Medicine Chicago Illinois USA; ^5^ Ann & Robert H. Lurie Children's Hospital of Chicago Chicago Illinois USA; ^6^ Department of Surgery University of Pittsburgh Medical Center Pittsburgh Pennsylvania USA; ^7^ John R. Oishei Children's Hospital and Jacobs School of Medicine and Biomedical Sciences, State University of New York University at Buffalo Buffalo New York USA; ^8^ US Department of Agriculture/Agricultural Research Service Children's Nutrition Research Center, Department of Pediatrics Baylor College of Medicine Houston Texas USA; ^9^ Department of Pediatric Surgery Nationwide Children's Hospital and The Ohio State University College of Medicine Columbus Columbus Ohio USA

## Abstract

**Objective:**

The long‐term durability of improvements in functional mobility and musculoskeletal pain for adolescents after metabolic and bariatric surgery (MBS) is unknown.

**Methods:**

We used the Teen‐Longitudinal Assessment of Bariatric Surgery (Teen‐LABS) study to determine the change in mobility and pain among adolescents who underwent MBS. From standardized 400‐m walk tests, we analyzed walk time, heart rate (HR) parameters, and musculoskeletal pain.

**Results:**

The mean walk time improved from 383 s (95% CI: 368–399) prior to surgery to 351 s (95% CI: 330–372) by 6 years. The mean resting HR was 90 beats per minute (bpm; 95% CI: 87–93) preoperatively and decreased to 80 bpm (95% CI: 76–84) by 6 years. The risk of any musculoskeletal pain decreased from 37.2% (95% CI: 25.5%–48.9%) to 11.0% (95% CI: 4.3%–17.6%) by 6 years. Mediation analysis revealed that the effect of time since surgery on walk time, resting HR, and HR recovery occurred through a weight‐dependent mechanism. For posttest HR and HR difference, there was both a significant weight‐dependent and weight‐independent mechanism. The effect of surgery on the risk of musculoskeletal pain occurred through a weight‐independent mechanism.

**Conclusions:**

Adolescents who underwent MBS experienced significant, durable improvement in mobility and pain, despite weight regain. Our models suggest that improvements may occur through a weight‐independent mechanism.

**Study Importance**

**What is already known?**

- Bariatric surgery is known to offer improvements in functional mobility and musculoskeletal pain in the short term, but the durability of the effects in the long term and in the setting of weight regain are unknown.

**What does this study add?**

- In this cohort study of adolescents who underwent bariatric surgery, improvements in functional mobility and musculoskeletal pain were sustained for up to 6 years after bariatric surgery, despite modest weight regain. These effects occurred through both weight‐dependent and weight‐independent mechanisms.

**How might these results change the direction of research or the focus of clinical practice?**

- Future research is needed to determine how bariatric surgery affects outcomes such as heart rate parameters and musculoskeletal pain through a weight‐independent mechanism.

## INTRODUCTION

Adolescents with severe forms of obesity, defined as class 2 obesity or higher (≥120% of 95th percentile or body mass index [BMI] ≥ 35 kg/m^2^), are at risk for significant limitations to functional mobility and musculoskeletal pain, especially in the lower extremities [1, 2, 3, 4, 5]. We and others have previously reported on the high prevalence of musculoskeletal pain and poor physical function among adolescents living with obesity [4, 6, 7]. Biomechanical differences between gait patterns and efficiency of high‐impact activities, such as running and jumping, between individuals with obesity and those with normal weight (BMI = 18.5–24.9) likely contribute to increased mechanical stress that increases the likelihood of musculoskeletal pain [8, 9].

Metabolic and bariatric surgery (MBS) improves functional mobility and musculoskeletal pain for up to 2 years in adolescents [1]. However, the durability of these improvements after 3 years in addition to potential long‐term relationships with postoperative weight change is presently unknown. Prior studies have proposed that the effects of MBS on mobility and pain outcomes may occur through both weight‐dependent and weight‐independent mechanisms, but there is little empirical evidence to substantiate the role of weight‐independent mechanisms. The relative contribution of both mechanisms is further complicated by weight regain after MBS, which has been well documented in the literature, with up to one‐quarter of adults who undergo MBS experiencing clinically significant weight regain from the postoperative nadir [10, 11, 12].

We investigated the functional mobility and probability of walking‐related musculoskeletal pain, as well as the relationship between these outcomes and weight regain, for up to 6 years after MBS among adolescents with severe obesity. As part of the Teen‐Longitudinal Assessment of Bariatric Surgery (Teen‐LABS) cohort study [13, 14], 400‐m walk tests were routinely administered at baseline, 6 months, and 1 year and annually for up to 6 years. Time to completion, resting heart rate (HR), immediate posttest HR, HR difference, HR recovery, and the risk of walking‐related musculoskeletal pain were assessed over the course of scheduled follow‐up research visits. We hypothesized that the improvements in functional mobility, cardiovascular parameters, and musculoskeletal pain observed postoperatively would be sustained at 6 years, even in the setting of weight regain, and that there would be a significant weight‐independent contribution to these outcomes.

## METHODS

### Study cohort and measurement points

Data for this analysis originated from the Teen‐LABS study [13, 14]. The design and analysis of the primary outcome at year 2 of follow‐up have been previously described [15]. Teen‐LABS is a prospective, multicenter, longitudinal, observational cohort study that enrolled adolescents who were 19 years of age or younger at the time of bariatric surgical intervention. Parental permission and either assent or consent of the children participating were obtained. The protocol and the data and safety monitoring plan were approved by the institutional review board at each participating institution and by the data and safety monitoring board for each study. The data used in the current analysis originate from the 400‐m walk tests conducted at baseline, at 6 months, and annually up to 6 years after the initial surgery. It should be noted that, although the 400‐m walk test is not a gold‐standard test like the graded‐exercise test, it was chosen because of its clinical utility and widespread use. At baseline, 205 participants completed a 400‐m walk test. At each follow‐up, height and weight measurements and body fat percentage were recorded using a wall‐mounted stadiometer and an electronic scale (Scale‐Tronix 5200, Welch Allyn, Inc., or TANITA TBF‐310, TANITA).

### Assessment of functional mobility

To measure functional mobility and cardiovascular fitness, time to complete the 400‐m walk test, resting HR, immediate posttest HR, HR difference between resting and immediate posttest HR, and recovery HR 2 min after the walk test were measured. HR was measured via a Polar HR monitor (Polar Electro, Inc.), and time to completion was measured with a stopwatch. Finally, musculoskeletal pain during the walk test was determined by participant report using a standardized survey instrument at each visit that has been previously described [4]. The survey collected information on the presence of numbness or tingling and leg cramps, as well as the severity of pain in the hip, knee, calf, foot, and back. These data were then used to create a composite outcome (any pain) for this analysis.

### Statistical analysis

Descriptive analyses were used for baseline characteristics of the cohort by type of bariatric surgery, consisting of either Roux‐en‐Y gastric bypass (RYGB) or vertical sleeve gastrectomy. We defined clinically significant weight regain as a BMI recorded after 12 months from surgery that was at least 20% greater than the documented 12‐month postoperative nadir at any point during follow‐up. Although the adult literature often proposes a cutoff of weight regain of 25%, there is significantly less consensus in the adolescent bariatric surgery literature. In order to favor capturing cases of true weight regain, we used a lower, but still clinically reasonable, threshold of 20% from the postoperative nadir [16, 17, 18, 19]. The covariates considered were time since surgery, age at the time of visit, sex, race and ethnicity, baseline BMI, enrollment site, type of surgery, and percent BMI change. We found that, in all models, type of surgery was not significantly associated with any outcomes and was therefore excluded from the primary models. We hypothesized that the effect of time since surgery on each outcome was mediated through BMI change, and we therefore excluded this variable from the primary model as adjustment for this mediator would obscure the relationship between time since surgery and each outcome. To obtain the estimated change in outcomes, we used statistical models that leveraged a mixed‐effects linear or logistic approach, while holding constant the variables of time since surgery, age at the time of the visit, sex, race and ethnicity (non‐Hispanic Black, non‐Hispanic White, Hispanic/Latino, or other), baseline BMI, and enrollment site. We included a random effect for each study participant to account for the within‐participant, between‐visit correlation. With respect to missing data, we estimated the mixed‐effects regression models using 10 imputations of missing outcomes assuming a multivariate normal function for walk time and HR outcomes and a logistic function for the composite outcome of musculoskeletal pain. The outcomes analyzed included total walk time in seconds, resting HR in beats per minute (bpm), immediate posttest HR in bpm, HR difference in bpm (immediate posttest HR − resting HR), HR recovery in bpm (posttest HR − 2‐min HR), and the composite binary outcome of any musculoskeletal pain.

For the mediation analysis, we used a set of two random‐effects linear regression models, adjusting for the between‐visit, within‐participant correlation. The first model was fit to model the functional mobility outcome, adjusting for visit, age, sex, race and ethnicity, baseline BMI, and site identifier. The second model included all terms of the prior model with the addition of a term capturing the percent BMI change, the hypothesized mediator, from the baseline BMI to the BMI at a given visit. The coefficients describing the change in the outcome at each visit were then compared between the model without BMI change and the model with BMI change. If the coefficients of the first model demonstrated a significant change in the functional mobility outcome by the last visit but there was no significant evidence for a change in the outcome by the last visit after adjusting for the change in BMI, and if the relationship between the change in BMI and functional outcome was significant, this provided evidence that the effect of time since surgery on the functional mobility outcome occurred through a weight‐dependent mechanism. If, after adjustment for the mediator, there was still a significant association between time since surgery and the outcome, and there was no association between the outcome and change in BMI, this suggested that there was a weight‐independent mechanism. Finally, if after adjustment there was still a significant, but smaller, association between time since surgery and the outcome, and there was an association between the outcome and change in BMI, this suggested that the effect of time since surgery on outcome occurred through both a weight‐dependent and weight‐independent mechanism.

We also used a supplemental causal mediation analysis to estimate the total causal effect, the natural indirect and direct effects, and the proportion of the relationship between time since surgery and outcome that is mediated by changes in BMI (proportion of mediation) [20]. Inferences were made with standard errors clustered at the level of site identifier. All analyses were completed with Stata version 18 (StataCorp LLC).

## RESULTS

In total, 205 study participants were included in this analysis, consisting of 146 (71.2%) who underwent RYGB and 59 (28.8%) who underwent vertical sleeve gastrectomy (Table 1). There was a moderate amount of incomplete data, with 185 (90.2%) participants at 6 months, 168 (82.0%) participants at 1 year, 143 (69.8%) participants at 2 years, 122 (59.5%) participants at 3 years, 125 (61.0%) participants at 4 years, 126 (61.5%) participants at 5 years, and 119 (58.0%) participants at 6 years who completed 400‐m walk tests. However, accounting for ineligibility due to dropping out of the study and completing remote visits that did not allow for 400‐m walk tests to be completed, there was an 86% completion rate at 6 months, 82% completion at 1 year, 79% completion at 2 years, 70% completion at 3 years, 73% completion at 4 years, 72% completion at 5 years, and 72% completion at 6 years. Of the participants, 154 (75.1%) were female, 131 (63.9%) were non‐Hispanic White, and 44 (21.5%) were non‐Hispanic Black. At baseline, the median age of the cohort was 17.1 years (IQR: 15.9–18.2 years) with a median body fat percentage of 53.2% (IQR: 50.2%–56.1%).

**TABLE 1:** Demographic and clinical characteristics of cohort.

	Total (*N* = 205)
Type of surgery, *n* (%)	
Gastric bypass	146 (71.2)
Sleeve gastrectomy	59 (28.8)
Sex, *n* (%)	
Male	51 (24.9)
Female	154 (75.1)
Race and ethnicity, *n* (%)	
Non‐Hispanic White	131 (63.9)
Non‐Hispanic Black	44 (21.5)
Hispanic/Latino	16 (7.8)
Other	14 (6.8)
Age, mean (SD), y	17.1 (15.9–18.2)
Body fat, mean (SD), %	53.2 (50.2–56.1)
BMI, mean (SD), kg/m^2^	50.5 (45.3–58.1)
Weight, mean (SD), kg	143.7 (127.6–165.6)
Height, mean (SD), cm	168.0 (162.2–174.0)
Weight regain, *n* (%)	
No regain	127 (62.0)
>20% regain	66 (32.2)
Visit completion with walk test, *n* (%)	
6 mo	185 (90.2)
1 y	168 (82.0)
2 y	143 (69.8)
3 y	122 (59.5)
4 y	125 (61.0)
5 y	126 (61.5)
6 y	119 (58.0)

Figure SA demonstrates the modeled BMI of participants with 95% confidence interval (CI) values across each visit. Prior to MBS, the mean BMI was 53.0 (95% CI: 51.9–54.4), which decreased to 36.7 (*p* < 0.001, 95% CI: 35.6–37.8) by the 1‐year visit, representing a 30.8% decrease in BMI. The mean BMI of the cohort then increased to 39.9 (*p* < 0.001, 95% CI: 38.2–41.6) by the 6‐year visit, with 66 (32.2%) participants experiencing clinically significant weight regain (>20%).

**FIGURE 1:**
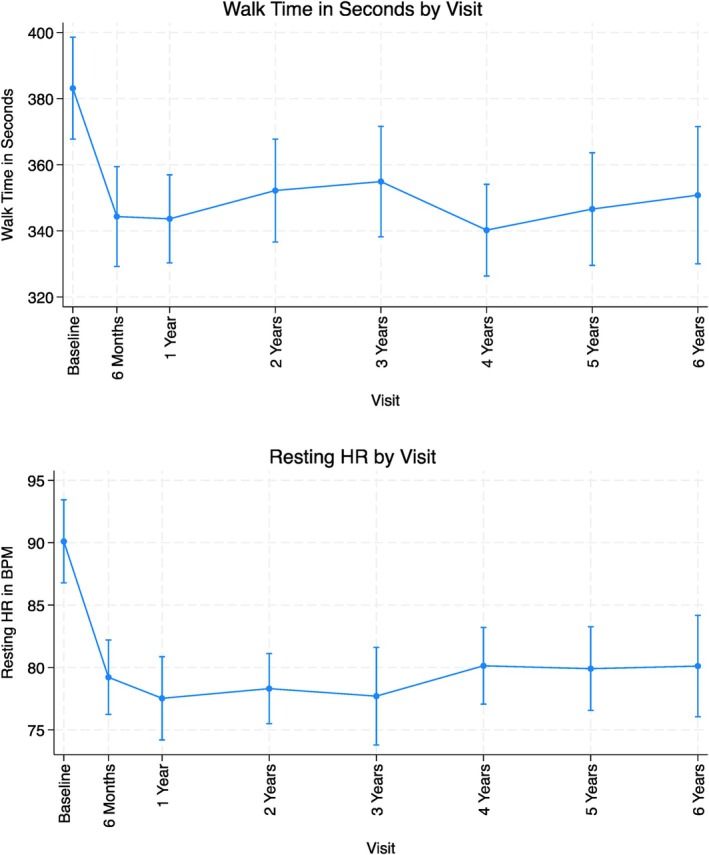
Walk time in seconds and resting HR in bpm by visit with 95% CI values. bpm, beats per minute; HR, heart rate. [Color figure can be viewed at wileyonlinelibrary.com]

The marginal estimates of walk time by visit are plotted in Figure 1. At baseline, the estimated average walk time for the cohort was 383 s (95% CI: 368–399), which decreased to 344 s (*p* < 0.001, 95% CI: 330–357) at 1 year and 351 s (*p* = 0.038, 95% CI: 330–372) by 6 years. Resting HR decreased from 90 bpm (95% CI: 87–93) to 78 bpm (*p* < 0.001, 95% CI: 74–81) at 1 year and was equal to 80 bpm (*p* = 0.003, 95% CI: 76–84) by 6 years.

**FIGURE 2:**
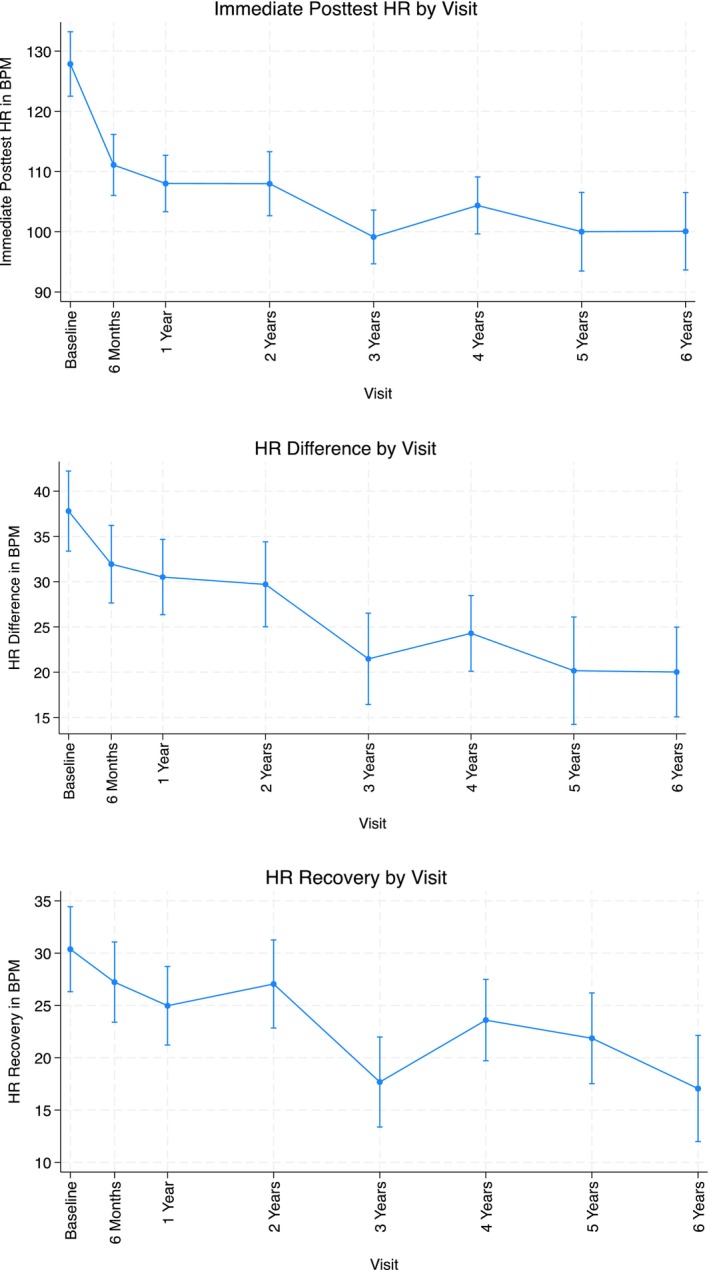
Immediate posttest HR, HR difference, and HR recovery by visit with 95% CI values. bpm, beats per minute; HR, heart rate. [Correction added on 21 May 2025, after first online publication: Figure 2 has been replaced for completeness.] [Color figure can be viewed at wileyonlinelibrary.com]

The marginal estimates of immediate posttest HR, HR difference, and 2‐min HR recovery estimated with a similar model structure are displayed by visit in Figure 2. Immediate posttest HR decreased from 128 bpm (95% CI: 123–133) at baseline to 108 bpm (*p* < 0.001, 95% CI: 103–113) at 1 year and 100 bpm (*p* < 0.001, 95% CI: 94–107) at 6 years. Similarly, the HR difference between the immediate posttest HR and the resting HR also decreased from 38 bpm (95% CI: 33–42) at baseline to 31 bpm (*p* = 0.003, 95% CI: 26–35) at 1 year and 20 bpm (*p* < 0.001, 95% CI: 15–25) at 6 years. HR recovery, that is, the difference between the immediate posttest HR and the 2‐min HR, also decreased from baseline at 30 bpm (95% CI: 26–34) to 25 bpm (*p* = 0.015, 95% CI: 21–29) at 1 year and 17 bpm (*p* = 0.001, 95% CI: 12–22) at 6 years.

**FIGURE 3:**
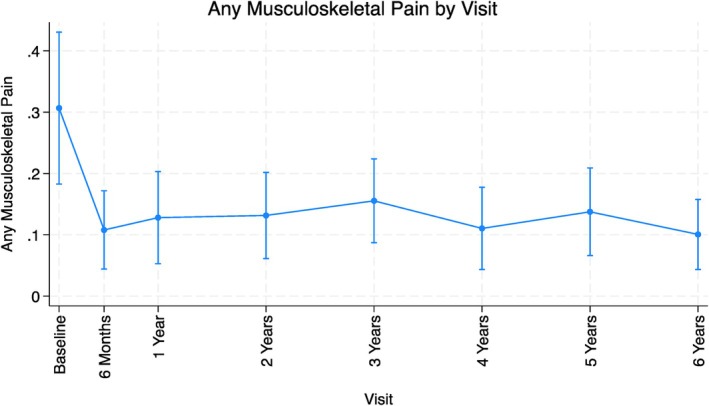
Modeled probability of any musculoskeletal pain by visit with 95% CI values. [Color figure can be viewed at wileyonlinelibrary.com]

The modeled prevalence of musculoskeletal pain, defined as the composite outcome of any pain in the hip, knee, back, calf, or foot, numbness or tingling, or leg cramps, by time since surgery is demonstrated in Figure 3. The probability of pain decreased from the baseline visit at 37.2% (95% CI: 25.5%–48.9%) to 14.1% (*p* = 0.003, 95% CI: 5.4%–22.8%) at 1 year and 11.0% (*p* = 0.014, 95% CI: 4.3%–17.6%) by 6 years. The prevalence of any musculoskeletal pain and each reported component of musculoskeletal pain by visit are displayed in Figure SB.

The mediation analysis revealed that, for the outcomes of walk time, resting HR, and HR recovery, prior to adjustment for BMI change, there were significant associations between the outcomes and time since surgery, and, after adjustment for BMI change, there was no longer an association between time since surgery and these outcomes, suggesting that the effect between these two occurred through a weight‐dependent mechanism (Table 2). For the outcomes of posttest HR and HR difference, there were significant associations between time since surgery both prior to and after adjustment for BMI change and an observed decrease in the coefficient size for time since surgery, suggesting that there was both a weight‐dependent and weight‐independent effect for these outcomes. Finally, for the outcome of musculoskeletal pain, there was a measurable effect of time since surgery both prior to and after adjustment for BMI change; however, there was no association between BMI change and the outcome, suggesting that the effect of time since surgery on musculoskeletal pain occurred through a weight‐independent mechanism. Except for HR recovery, the conclusions of the supplemental causal mediation analysis were consistent with the primary analysis (Table S1).

**TABLE 2:** Multivariate logistic model for functional mobility outcomes before and after adjustment for percent change in BMI.

	Walk time (s)	Walk time (s) after adjustment for BMI change	Resting HR (bpm)	Resting HR (bpm) after adjustment for BMI change	Posttest HR (bpm)	Posttest HR (bpm) after adjustment for BMI change	HR difference (bpm)	HR difference (bpm) after adjustment for BMI change	HR recovery (bpm)	HR recovery (bpm) after adjustment for BMI change	Probability of musculoskeletal pain	Probability of musculoskeletal pain after adjustment for BMI change
Time since surgery (Ref: baseline visit)												
6 y	−32.3* (−62.8 to −1.9)	−2.1 (−33.6 to 29.4)	−10.0** (−16.5 to −3.4)	−4.6 (−11.2 to 2.1)	−27.8*** (−38.0 to −17.6)	−15.1* (−26.8 to −3.4)	−17.8*** (−25.6 to −10.0)	−10.7* (−19.6 to −1.7)	−13.3** (−21.3 to −5.3)	−6.4 (−15.2 to 2.4)	0.1** (0.0 to 0.60)	0.2* (0.0 to 0.7)
Percent BMI change		−130.1*** (−178.9 to −81.3)		−23.1*** (−34.8 to −11.3)		−54.8*** (−77.9 to −31.7)		−30.9** (−48.5 to −13.3)		−29.9*** (−45.6 to −14.2)		0.3 (0.0 to 1.6)
Age	−2.1 (−5.9 to 1.8)	−1.9 (−5.6 to 1.9)	0.0 (−0.9 to 1.0)	0.1 (−0.8 to 1.0)	0.5 (−0.9 to 1.9)	0.6 (−0.7 to 1.9)	0.4 (−0.6 to 1.5)	0.5 (−0.5 to 1.5)	0.2 (−0.8 to 1.2)	0.3 (−0.7 to 1.2)	1.3* (1.1 to 1.5)	1.3* (1.0 to 1.5)
Sex (Ref: male)												
Female	8.2 (−5.1 to 21.6)	2.8 (−10.3 to 15.9)	2.6 (−0.4 to 5.5)	1.6 (−1.4 to 4.6)	10.1*** (5.8 to 14.5)	7.8*** (3.4 to 12.2)	7.5*** (4.1 to 11.0)	6.2*** (2.7 to 9.8)	6.1*** (3.0 to 9.3)	4.9** (1.7 to 8.1)	1.9* (1.1 to 3.3)	1.9* (1.1 to 3.5)
Race and ethnicity (Ref: Non‐Hispanic White)												
Non‐Hispanic Black	12.4 (−2.2 to 27.0)	8.4 (−5.8 to 22.6)	−0.4 (−3.7 to 3.0)	−1.1 (−4.4 to 2.2)	0.7 (−4.4 to 5.7)	−1.0 (−5.9 to 3.8)	1.0 (−3.3 to 5.2)	0.0 (−4.1 to 4.1)	−0.1 (−3.7 to 3.6)	−1.0 (−4.5 to 2.5)	0.6 (0.3 to 1.2)	0.5 (0.3 to 1.0)
Hispanic/Latino	−1.7 (−25.4 to 22.1)	−2.2 (−25.4 to 21.0)	−2.8 (−8.1 to 2.5)	−2.9 (−8.1 to 2.3)	−2.2 (−10.5 to 6.1)	−2.4 (−10.4 to 5.7)	0.7 (−6.4 to 7.9)	0.6 (−6.5 to 7.7)	0.5 (−5.4 to 6.5)	0.4 (−5.6 to 6.4)	0.4 (0.1 to 1.1)	0.3* (0.1 to 0.9)
Other	−0.5 (−24.8 to 23.7)	−4.4 (−27.7 to 19.0)	−1.1 (−6.4 to 4.1)	−1.8 (−7.0 to 3.4)	−5.9 (−14.3 to 2.5)	−7.6 (−15.6 to 0.5)	−4.7 (−12.3 to 2.8)	−5.7 (−12.9 to 1.6)	−7.2* (−13.2 to −1.3)	−8.1** (−13.9 to −2.4)	0.7 (0.2 to 2.0)	0.6 (0.2 to 2.0)
Baseline BMI, kg/m^2^	2.3*** (1.6 to 3.0)	2.1*** (1.5 to 2.8)	0.2* (0.0 to 0.3)	0.2* (0.0 to 0.3)	0.6*** (0.4 to 0.9)	0.6*** (0.3 to 0.8)	0.5*** (0.3 to 0.7)	0.4*** (0.2 to 0.6)	0.3*** (0.2 to 0.5)	0.3*** (0.1 to 0.5)	1.1*** (1.0 to 1.1)	1.1*** (1.0 to 1.1)

*Note*: 95% CI values in brackets.

Abbreviations: bpm, beats per minute; HR, heart rate; Ref, reference.

* *p* < 0.05.

** *p* < 0.01.

*** *p* < 0.001.

## DISCUSSION

Our findings suggest that the effects of MBS on functional mobility and musculoskeletal pain are durable for up to 6 years. Improvements in walk time and resting HR were sustained to a similar level between 6 months and up to 6 years after surgery. Steady improvements in immediate posttest HR, HR difference, and HR recovery occurred over time, suggesting that, although the initial improvements in these parameters may be due to surgical intervention, other factors such as decreased inflammatory stress and metabolic adaptations may be contributing to sustained improvement in these proxies for cardiovascular fitness. Notably, immediate posttest HR, HR difference, and musculoskeletal pain were identified as having a significant weight‐independent mechanism through the mediation analysis. Taken together, we demonstrate sustained improvements in mobility, HR response to exercise, and musculoskeletal pain, despite modest, significant weight regain, suggesting a significant role of the weight‐independent effect of surgery on these outcomes.

Weight regain after MBS has been well described in the adult and adolescent literature [10, 11, 12]. Evidence suggests that, after 6 years of follow‐up, up to 37% of individuals who underwent bariatric surgery experienced weight regain, defined as at least 25% weight regain from the nadir [21]. We observed a similar prevalence of weight regain in our study, in which 66 (34.2%) individuals in the cohort had a BMI after 1 year after operation that was 20% greater than the nadir, defined as the lowest BMI recorded within 1 year after surgery.

Although some previous studies have also documented recurrence of musculoskeletal pain after MBS in the context of weight regain, it is unclear whether this is the inherent result of weight gain or whether pain increased the difficulty in sustaining post‐surgery lifestyle changes [22]. Nevertheless, our work has previously shown that the protective effect of MBS on musculoskeletal pain occurs for up to 2 years [1]. Our current study extends this evidence out to 6 years by demonstrating that the risk of musculoskeletal pain remained persistently decreased over the course of 6 years of follow‐up and that this improvement occurs through a significant weight‐independent mechanism. This is consistent with the durability of improvements in pain and physical function reported in adults for up to 7 years after bariatric surgery [23]. When compared with adults with similar pre‐surgery characteristics, adults who had undergone RYGB exhibited a lower risk of mobility difficulty for at least 5 years after surgery [24].

Taken together, the sustained benefits in functional mobility and musculoskeletal pain were observed in this cohort for up to 6 years after surgery, despite more than one‐quarter of the cohort increasing their BMI by more than 20% after the initial nadir after surgery. Some parameters such as walk time, resting HR, and any musculoskeletal pain were found to have sustained a similar level of improvement between 6 months and 6 years, whereas others such as posttest HR, HR difference, and HR recovery were observed to continue to improve over time.

The mediation analysis revealed that, for all outcomes except for walk time and resting HR, a significant portion of the effect of surgery on the outcome occurred through a weight‐independent mechanism, and we speculate that this pathway may be responsible for driving the observed continued improvement in some of these outcomes. This is consistent with work by Aminian et al., who leveraged a propensity score analysis to match patients who underwent MBS to nonsurgical patients receiving standard care and found that the positive effects of MBS were present even after adjusting for weight loss [25]. Although the exact mechanism is unclear, the Utah Obesity Study additionally found that there were favorable adaptations to cardiac morphology following MBS, which may also contribute to the sustained improvement in cardiovascular fitness that was observed in our study. This hypothesis requires further study in a cohort designed to answer the weight‐dependent and weight‐independent outcomes.

**FIGURE 4:**
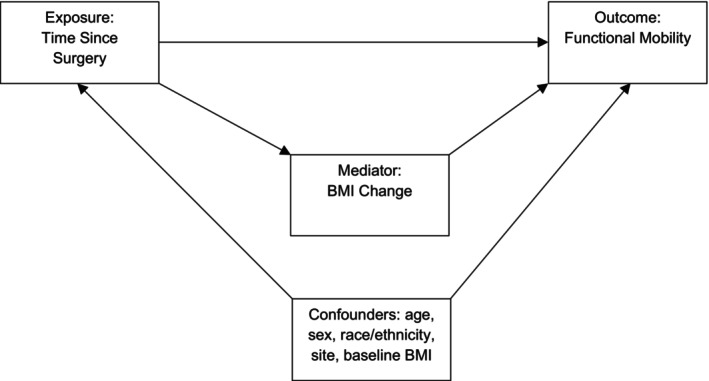
Directed acyclic graph of the relationship between weight and functional mobility outcome.

There were several strengths and limitations to this study. First, this cohort provides access to longitudinal data as well as high‐fidelity measures of cardiovascular fitness for a large sample of patients who underwent MBS as adolescents. Although there was a moderate degree of attrition, we leveraged imputation models that provided robust estimates even under varied specifications. This study was limited by a lack of a nonsurgical comparator group, which would have helped estimate the counterfactual functional mobility and pain outcomes had these patients not received MBS. Furthermore, model misspecification, unmeasured or residual confounding, and misclassification of outcomes all may threaten the validity of these conclusions. Finally, given that subsequent visits after the first year of follow‐up occurred in 1‐year intervals, more frequent follow‐up data may have provided a more granular view of the changes to functional mobility and walking‐related musculoskeletal pain (Figure 4).

## CONCLUSION

In a prospective, longitudinal cohort of adolescents with severe obesity who underwent MBS, we found that the effects of improved functional mobility, cardiovascular parameters, and musculoskeletal outcomes were sustained or continued to improve until 6 years of follow‐up. These improvements were observed despite modest, clinically significant weight regain and occurred through both weight‐dependent and weight‐independent mechanisms.

## FUNDING INFORMATION

Funding for Teen‐LABS was provided by the National Institutes of Health (NIH) (U01DK072493 / UM1 DK072493 to T.H.I.) (UM1 DK095710 to C.X., T.M.J.) and the National Center for Research Resources and the National Center for Advancing Translational Sciences, NIH (8UL1TR000077). Support also came from National Center for Research Resources and the National Center for Advancing Translational Sciences, NIH, (UL1TR000114).

## CONFLICT OF INTEREST STATEMENT

Justin R. Ryder receives support from Boehringer Ingelheim Pharmaceuticals in the form of drugs and placebos. Stephanie Sisley has received speaking and consulting fees from Rhythm Pharmaceuticals that are unrelated to this project. Marc P. Michalsky has received educational honoraria from Intuitive Surgical, Inc., and serves on a pediatric obesity advisory panel for Eli Lilly and Company, both unrelated to this project. Thomas H. Inge has served as consultant for Standard Bariatrics and Independent Medical Expert Consulting Services, both unrelated to this project. Anita P. Courcoulas has research support from Allurion Technologies and Eli Lilly and Company. The other authors declared no conflicts of interest.

## Supporting information


**Table S1.** Total causal effects, natural direct and indirect effects, and proportion of mediation between percent BMI change from baseline and functional mobility or musculoskeletal pain outcomes.

**Figure SA.** BMI by visit with 95% confidence intervals.

**Figure SB.** Prevalence of any (red) and no (blue) musculoskeletal pain and components by visit.
