# Supplementary material for: Functional mobility and pain are improved for 6 years after adolescent bariatric surgery
Source: Obesity (Silver Spring). 2025 Apr 21;33(6):1126–35. doi: 10.1002/oby.24285 (PMC12119214; doi:10.1002/oby.24285)
Supplement: Supplementary file 1 — Table S1. Total causal effects, natural direct and indirect effects, and proportion of mediation between percent BMI change from baseline and functional mobility or musculoskeletal pain outcomes. [file OBY-33-1126-s002.docx]

**Supplemental Figure A. BMI by Visit with 95% Confidence Intervals**
