# Supplementary material for: Functional mobility and pain are improved for 6 years after adolescent bariatric surgery
Source: Obesity (Silver Spring). 2025 Apr 21;33(6):1126–35. doi: 10.1002/oby.24285 (PMC12119214; doi:10.1002/oby.24285)
Supplement: Supplementary file 2 — Figure SA. BMI by visit with 95% confidence intervals. [file OBY-33-1126-s003.docx]

**Supplemental Figure B. Prevalence of Any (Red) and No (Blue) Musculoskeletal Pain and Components by Visit**
