# Supplementary material for: Functional mobility and pain are improved for 6 years after adolescent bariatric surgery
Source: Obesity (Silver Spring). 2025 Apr 21;33(6):1126–35. doi: 10.1002/oby.24285 (PMC12119214; doi:10.1002/oby.24285)
Supplement: Supplementary file 3 — Figure SB. Prevalence of any (red) and no (blue) musculoskeletal pain and components by visit. [file OBY-33-1126-s001.docx]

**Supplemental Table 1. Total causal effects, natural direct and indirect effects, and proportion of mediation between percent BMI change from baseline and functional mobility or musculoskeletal pain outcomes**

| Variable Name | Total Causal Effect (95% CI) | Weight-Independent Effect (95% CI) | Weight-Independent Contribution % (95% CI) | Weight-Dependent  Effect (95% CI) | Weight-Dependent Contribution % (95% CI) |
| --- | --- | --- | --- | --- | --- |
| Walk Time (seconds) | -27.1 (-46.7, -7.53) | 0.0 (-20.7, 20.7) | 0.0 (0.0, 76.5) | -27.1 (-36.1, -18.1) | 100.0 (23.5, 100.0) |
| Resting HR (bpm) | -7.9 (-11.0, -4.8) | -2.1 (-5.5, 1.2) | 27.1 (0.0, 61.6) | -5.7 (-7.7, -3.8) | 72.9 (38.4, 100.0) |
| Immediate Posttest HR (bpm) | -26.7 (-31.9, -21.4) | -17.2 (-23.1, -11.4) | 64.6 (50.8, 78.5) | -9.4 (-12.9, -6.0) | 35.4 (21.6, 49.2) |
| HR Difference (bpm) | -19.1 (-23.6, -14.5) | -15.5 (-20.5, -10.5) | 81.4 (68.2, 94.5) | -3.6 (-6.0, -1.1) | 18.6 (5.5, 31.8) |
| HR Recovery (bpm) | -13.6 (-18.2, -9.1) | -9.7 (-14.4, -4.9) | 71.0 (54.1, 87.9) | -4.0 (-6.0, -1.9) | 29.0 (12.1, 45.9) |
| Probability of Any Musculoskeletal Pain | -0.29 (-0.40, -0.20) | -0.27 (-0.36, -0.17) | 92.1 (78.7, 100.0) | -0.02 (-0.06, 0.02) | 7.9 (0, 21.3) |
